# Silica nanoparticles enhance autophagic activity, disturb endothelial cell homeostasis and impair angiogenesis

**DOI:** 10.1186/s12989-014-0050-8

**Published:** 2014-09-30

**Authors:** Junchao Duan, Yongbo Yu, Yang Yu, Yang Li, Peili Huang, Xianqing Zhou, Shuangqing Peng, Zhiwei Sun

**Affiliations:** School of Public Health, Capital Medical University, Beijing, 100069 P.R. China; Beijing Key Laboratory of Environmental Toxicology, Capital Medical University, Beijing, 100069 P.R. China; Institute of Disease Control and Prevention, Academy of Military Medical Sciences, Beijing, 100071 P.R. China

**Keywords:** Silica nanoparticles, Autophagy, Endothelium, Angiogensis, VEGFR2, Crosstalk, Nanotoxicology

## Abstract

**Background:**

Given that the effects of ultrafine fractions (<0.1 μm) on ischemic heart diseases (IHD) and other cardiovascular diseases are gaining attention, this study is aimed to explore the influence of silica nanoparticles (SiNPs)-induced autophagy on endothelial cell homeostasis and angiogenesis.

**Methods and results:**

Ultrastructural changes of autophagy were observed in both vascular endothelial cells and pericytes in the heart of ICR mice by TEM. Autophagic activity and impaired angiogenesis were further confirmed by the immunohistochemistry staining of LC3 and VEGFR2. In addition, the immunohistochemistry results showed that SiNPs had an inhibitory effect on ICAM-1 and VCAM-1, but no obvious effect on E-selectin in vivo. The disruption of F-actin cytoskeleton occurred as an initial event in SiNPs-treated endothelial cells. The depolarized mitochondria, autophagic vacuole accumulation, LC3-I/LC3-II conversion, and the down-regulation of cellular adhesion molecule expression were all involved in the disruption of endothelial cell homeostasis in vitro. Western blot analysis indicated that the VEGFR2/PI3K/Akt/mTOR and VEGFR2/MAPK/Erk1/2/mTOR signaling pathway was involved in the cardiovascular toxicity triggered by SiNPs. Moreover, there was a crosstalk between the VEGFR2-mediated autophagy signaling and angiogenesis signaling pathways.

**Conclusions:**

In summary, the results demonstrate that SiNPs induce autophagic activity in endothelial cells and pericytes, subsequently disturb the endothelial cell homeostasis and impair angiogenesis. The VEGFR2-mediated autophagy pathway may play a critical role in maintaining endothelium and vascular homeostasis. Our findings may provide experimental evidence and explanation for cardiovascular diseases triggered by nano-sized particles.

**Electronic supplementary material:**

The online version of this article (doi:10.1186/s12989-014-0050-8) contains supplementary material, which is available to authorized users.

## Background

With the development of nanotechnology, the engineered nanomaterials (ENMs) are becoming a significant part of the material flows in the global economy [1]. Silica nanoparticles (SiNPs) are one of the most widely applied engineered nanomaterials. The sources of SiNPs are very complicated, including natural nanoparticles and engineered nanoparticles [2,3]. A recent assessment on the global life cycle of ENMs showed that a significant fraction go into the soil (8–28%), water (0.4-7%), and atmosphere (0.2-1.5%). Among them, SiNPs are the dominant components in the global life cycle of ENMs [4]. There are multiple exposure pathways to SiNPs, such as the occupational exposure to SiNPs in the process of production, environmental exposure to SiNPs from air pollution, combustion process and indoor air pollution [5,6]. More recently, the iatrogenic exposure to SiNPs (commonly as carrier for delivery system) has become the important exposed ways in biomedical fields [7,8]. Since SiNPs are mainly used in gene therapy and drug delivery system, the circulation system could be directly exposed to SiNPs intravenously, and it is possible to cause harmful effects on cardiovascular system. Nano-sized silica is on the lists for toxicity evaluation by the National Institute of Environmental Health Sciences (NIEHS) and the Organisation for Economic Co-operation and Development (OECD) [9,10]. Therefore, it is meaningful to study the effects of SiNPs on cardiovascular toxicity.

As the environment and ecological system are always being in a dynamic recycling process, it is important to understand how ENMs affect human health and environmental safety. Since natural nanoparticles and engineered nanoparticles are becoming an important part of environmental particulate matters (PM), more than 99% of the total concentration of particles in the atmospheric environment is smaller than 300 nm [11]. Evidence from worldwide epidemiological studies confirm that the increased risk for mortality attributed to PM_2.5_ exposure is greater for cardiovascular than pulmonary diseases [12]. The 2010 American Heart Association (AHA) scientific statement has already reached several new conclusions, especially on the evidence that exposure to elevated levels of PM_2.5_ is strongly linked with ischemic heart diseases (IHD); yet, the toxicological mechanisms for the cardiovascular effects of ultrafine (<0.1 μm) particles are still unclear [13,14]. Given that the ENMs are inevitably transferred to the atmospheric environment and their sizes are within the range of ultrafine particles (UFPs), it is reasonable to use ENMs as a nano-sized model for clarifying the cardiovascular effects.

Currently, study on the biological behavior of ENMs in endothelial cells is important for safety evaluation of ENMs. In the 2013 OECD final report entitled “Safety of Manufactured Nanomaterials”, the transformation, degradation and persistence of ENMs have received the highest priority, and the degradation of ENMs can influence biological distribution and behavior [15]. Autophagy, a highly regulated cellular process for degrading proteins or organelles and the subsequent recycling of cellular products, plays an essential role in maintaining cellular homeostasis [16]. In contrast, excessive induction of the autophagic process is emerging as a potential mechanism of nanomaterial toxicity and contributes to disease pathogenesis [17,18]. Recent studies indicate that autophagy is involved in the angiogenic behavior of endothelial cells [19-21]. Angiogenesis is closely associated with the pathological progression of several cardiovascular diseases, including IHD, ischemic limb disease (ILD) and peripheral vascular disease (PVD) [22]. However, it remains unknown on how ENMs-induced autophagy may affect endothelial cell homeostasis and angiogenesis.

In our previous studies, we evaluated the acute toxicity of intravenously administrated SiNPs in vivo [23], and confirmed that SiNPs induced autophagy and autophagic cell death in vitro [24]; meanwhile, SiNPs had an inhibitory effect on angiogenesis of zebrafish via the down-regulation of VEGFR2/Erk1/2 signaling pathway [25]. This study is an extension of our previous study, which s aimed at fully understanding the effects and interaction mechanisms of SiNPs-induced autophagy on endothelial cell homeostasis and angiogenesis. The present study can provide more persuasive evidence for safety evaluation and risk management of nanomaterials.

## Results

### Characterization of SiNPs

**Figure 1:**
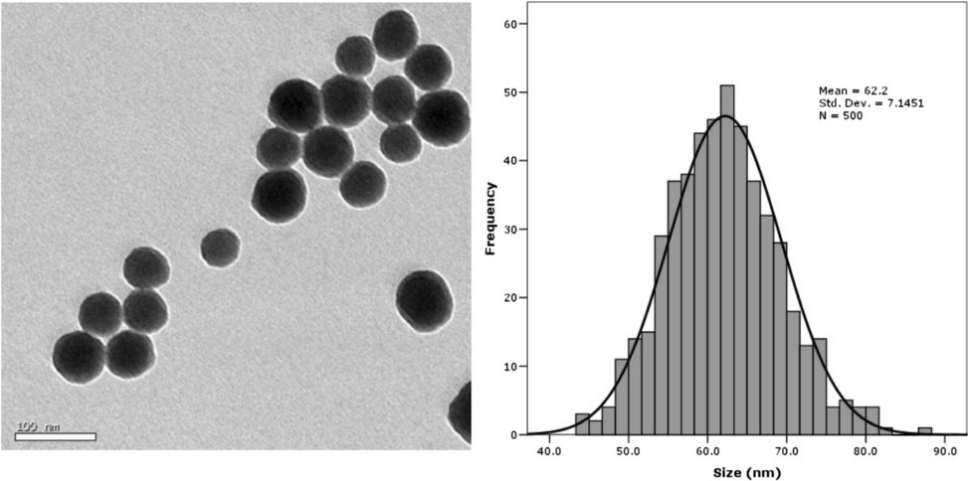
**Characterization of SiNPs.** The TEM images of SiNPs exhibited near-spherical shape with relatively favorable dispersibility. The size distribution showed that the average diameter of SiNPs was approximately 62 nm.

The transmission electron microscope (TEM) images of SiNPs exhibited a near-spherical shape with relatively favorable dispersibility (Figure 1). The size distribution showed that the average diameter of SiNPs was approximately 62 nm. The hydrodynamic sizes and Zeta potentials of SiNPs at different time points were detected in a stock media of distilled water, and in culture media of Dulbecco’s Modified Eagle’s Medium (DMEM) and physiological saline (Additional file 1: Table S1). The purity of SiNPs was more than 99.9% with no detectable endotoxin. Our results demonstrated that the SiNPs possessed good purity, monodispersity and stability in culture medium.

### *In vivo* study

#### Ultrastructural changes of SiNPs-induced autophagy in heart tissue

**Figure 2:**
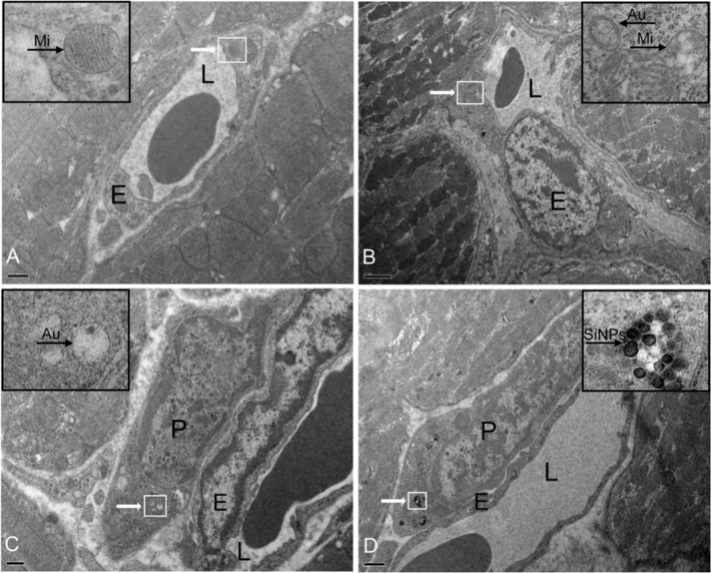
**TEM image of heart in ICR mice after acute exposure to SiNPs. (A)** Control group; **(B)** Numerous free ribosomes, autophagic vacuoles (black arrow), and swollen mitochondria (black arrow) were observed in 177.5 mg/kg SiNPs-treated group; **(C)** Several autophagic vacuoles (black arrow) were found in pericytes in 177.5 mg/kg SiNPs-treated group; **(D)** Localization of internalized SiNPs (black arrow) in 177.5 mg/kg SiNPs-treated group. White arrows, denote the original regions which are magnified on the upper corner; Scale bar: **(A)** 0.5 μm; **(B)** 2 μm; **(C)** 0.5 μm; **(D)** 1 μm. Abbreviation: E, endothelial cell; P, pericyte; L, lumen; Au, autophagic vacuoles; Mi, mitochondria.

The TEM images showed that the autophagic vacuoles, numerous free ribosomes, and swollen mitochondria with rupturing or disappeared cristae were presented in endothelial cells in SiNPs-treated group compared to control group (Figures 2A, 2B). Interestingly, we also found that some of the pericytes had several autophagic vacuoles in them (Figure 2C), and the SiNPs were internalized into pericytes (Figure 2D). The activation of SiNPs-induced autophagy occurred not only in endothelial cells, but also in pericytes.

#### Influence of SiNPs on autophagy, apoptosis and angiogenesis in heart tissue

**Figure 3:**
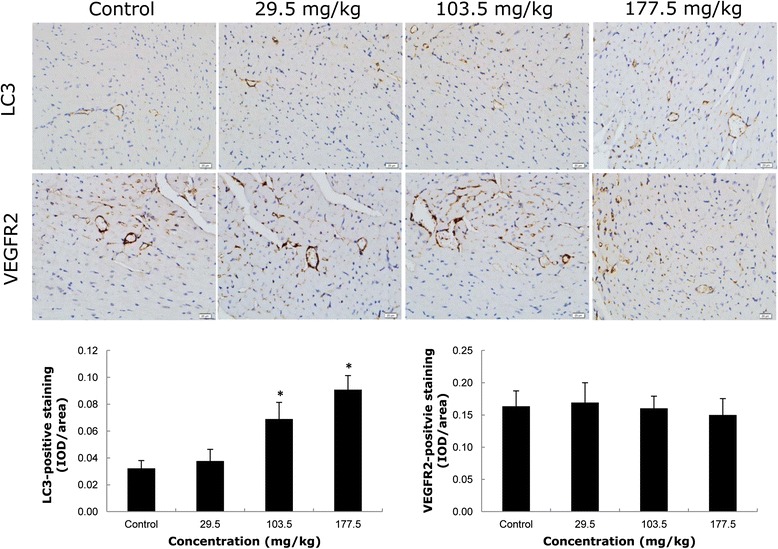
**Immunohistochemistry of LC3, VEGFR2 staining in ICR mice heart tissue sections.** Both LC3 and VEGFR2 positive cells were mainly located in the vascular endothelium rather than in cardiomyocytes. The LC3 positive cells increased in a dose-dependent manner while the VEGFR2 positive cells decreased in SiNPs-treated groups. IOD/area: Integrated optical density per stained area. *p < 0.05 compared with control group using ANOVA. Data are representative of at least six mice.

The microtubule-associated protein 1 light chain 3β (MAP1LC3B, or LC3) and vascular endothelial growth factor receptor 2 (VEGFR2) were measured in heart tissue sections using immunohistochemistry. As shown in Figure 3, both LC3 and VEGFR2 positive cells were mainly located on the vascular endothelium instead of cardiomyocytes. The staining of LC3 positive cells in the treatment groups was more intensive than that in the control group. In the intermediate (103.5 mg/kg) and high (177.5 mg/kg) dosage groups of SiNPs, the LC3 positive cells were significantly elevated (2.14- and 2.81- fold higher than that in control, respectively). Although there was no significant difference between the control group and the SiNPs-treated groups, the number of VEGFR2 positive cells decreased gradually in all SiNPs-treated groups. The apoptosis in heart tissue was further measured by the terminal deoxynucleotidyl transferase (TdT)-mediated dUTP nick end labeling (TUNEL) assay. There was no detectable expression of TUNEL positive cells in any treatment group (Additional file 1: Figure S1). In addition, no marked morphological change was observed in the SiNPs-treated groups by histopathological examination (Additional file 1: Figure S1).

#### Effect of SiNPs on cellular adhesion molecule expression in heart tissue

**Figure 4:**
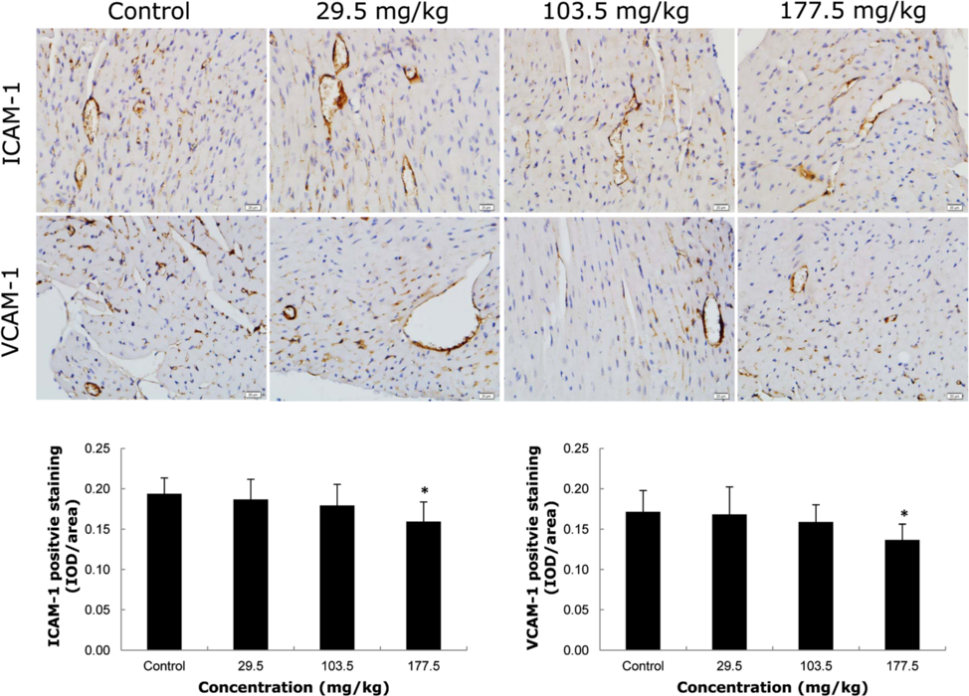
**Effects of SiNPs on cellular adhesion molecule expression in ICR mice heart tissue sections.** The ICAM-1 and VCAM-1 expression were decreased significantly compared to control in a dose-dependent manner. IOD/area: Integrated optical density per stained area. *p < 0.05 compared with control group using ANOVA. Data are representative of at least six mice.

As shown in Figure 4, the results of immunohistochemistry showed that both the vascular cell adhesion molecule-1 (VCAM-1) and intercellular adhesion molecule-1 (ICAM-1) were mainly expressed on vascular endothelium rather than cardiomyocytes. The expressions of cellular adhesion molecule (ICAM-1, VCAM-1) were not obviously different between the low (29.5 mg/kg) and middle (103.5 mg/kg) dosage groups and the control group. However, both ICAM-1 and VCAM-1 expression were reduced significantly in the high (177.5 mg/kg) dosage group compare to the control group. In addition, a weak expression of endothelial selectin (E-selectin) was detected on endothelium, and there was no significant difference between the control and SiNPs-treated groups (Additional file 1: Figure S1). These results indicated that SiNPs had an inhibitory effect on the expression of ICAM-1 and VCAM-1, but had no obvious effect on E-selectin expression.

### *In vitro* study

#### Effect of SiNPs on cellular uptake and cytoskeleton organization in HUVECs

**Figure 5:**
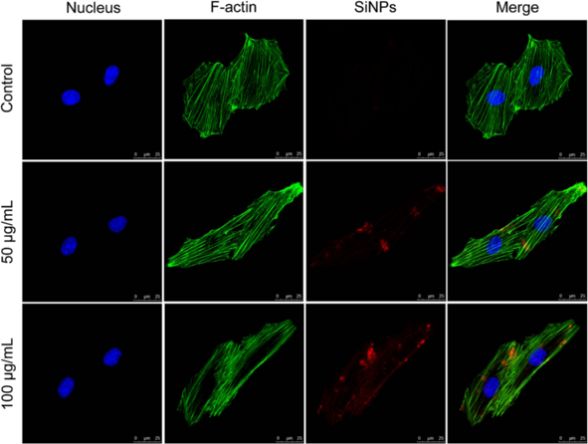
**Effect of SiNPs on cellular uptake and cytoskeleton organization.** The cytoskeletal F-actin in HUVECs were stained with Phalloidin-FITC Actin-Tracker(Green) and the cell nucleus with 4,6-diamidino-2-phenylindole, DAPI (blue) after treatment with Ruthenium (II) hydrate (Ru(phen)^32+^) interior-labeled SiNPs (red). The red fluorescent-labeled SiNPs entered into the HUVECs and disrupted the cytoskeleton F-actin in SiNPs-treated groups.

The effects of SiNPs on cellular uptake and cytoskeleton organization in the primary human umbilical vein endothelial cell line (HUVECs) were measured by laser scanning confocal microscopy (LSCM) (Figure 5). The red fluorescent-labeled SiNPs entered into the HUVECs in a dose-dependent manner. In the control group, there was a number of well organized filamentous actin (F-actin) in thick bundles in the cellular cytoplasm. However, with the dosage elevated, the F-actin in SiNPs-treated HUVECs was disorganized and disrupted. In the high (100 μg/mL) group of SiNPs, the structure of F-actin was non-isotropically assembled and the fluorescent intensity of F-actin was weakened compared to control group. The results demonstrated that SiNPs could disrupt the cytoskeleton organization in endothelial cells.

#### SiNPs-induced mitochondrial depolarization and autophagy in HUVECs

**Figure 6:**
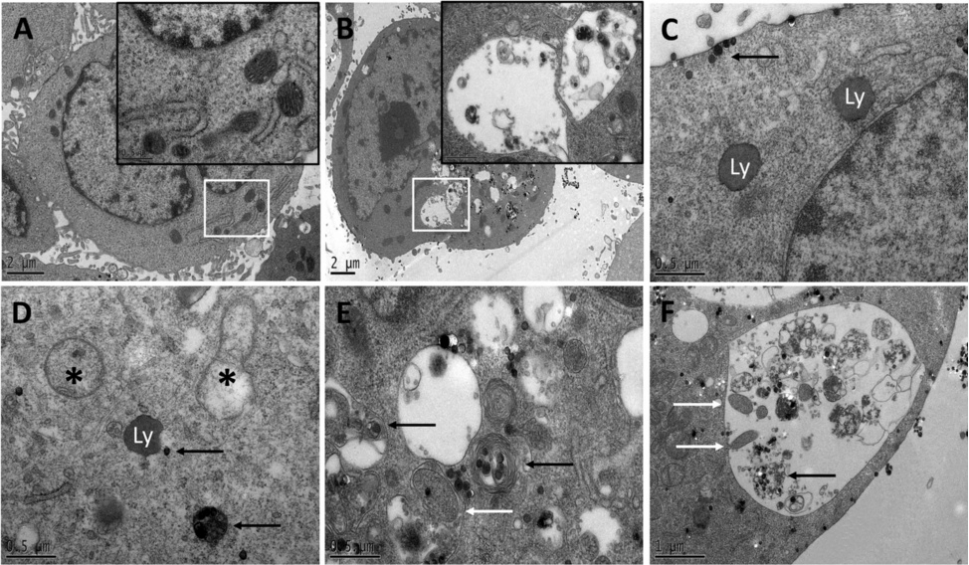
**Mitochondrial damage and autophagic activity triggered by SiNPs. (A)** Untreated cells. **(B)** Typical autophagic vacuoles with partially degraded cytoplasmic materials and electron-dense SiNPs. The whole process of SiNPs-mediated autophagy in HUVECs as follow: **(C)** Electron-dense SiNPs (black arrow) were internalized into cells via endocytic pathways; **(D)** SiNPs (black arrow) dispersed in cytoplasm either free or as membrane-bound aggregates in lysosomes, accompanyied with swollen mitochondria (asterisk); **(E)** Autolysosomes with double-layered membranes contained cellular debris, some of them contained mitochondria (white arrow) or electron-dense SiNPs (black arrow) were undergoing degradation at different stages; **(F)** an obviously larger autophagic vacuole was formed after vesicle fusion containing mitochondria (white arrow) and electron-dense SiNPs (black arrow).

Mitochondrial membrane potential (MMP), a sign of damage to mitochondrial membrane, was measured by a fluorescent probe 5,5’,6,6’-tetrachloro-1,1’,3,3’-tetraethylbenzimi dazo-lylcarbocyanide iodine (JC-1). The alteration of MMP was expressed by the ratio of green/red fluorescence intensity. With the dosage increasing, more loss of MMP was caused by SiNPs (Additional file 1: Figure S2). In addition, damaged mitochondria and activated autophagy were observed by ultrastructural analysis (Figure 6). As shown in Figure 6B, HUVECs treated with SiNPs displayed typical autophagic vacuoles with partially degraded cytoplasmic materials and electron-dense SiNPs compared to untreated cells (Figure 6A). Several other ultrastructures were observed as a part of the whole process of SiNP-mediated autophagy in HUVECs: the electron-dense SiNPs were internalized into cells through endocytic pathways (Figure 6C); SiNPs dispersed in cytoplasm either free or as membrane-bound aggregates in lysosomes with swollen or cristae-rupturing mitochondria (Figure 6D). Autolysosomes with double-layered membranes contained cellular debris, and those containing mitochondria or electron-dense SiNPs were in different stages of degradation (Figure 6E); an obviously larger autophagic vacuole was formed after vesicle fusion (Figure 6F). The SiNPs-induced autophagy was further verified by assessing the LC3-I/LC3-II conversion. The western blot analysis showed that the LC3-II/LC3-I ratio was significantly elevated, suggesting that the autophagic activity was enhanced by SiNPs in a dose-dependent manner (Additional file 1: Figure S3).

#### Effect of SiNPs on cellular adhesion molecule expression in HUVECs

To investigate the impact of SiNPs on cellular adhesion, the expressions of ICAM-1, VCAM-1 and E-selectin were measured by western blot analysis (Additional file 1: Figure S4). In the low dosage group (25 μg/mL), there was no marked change in the expression of ICAM-1 and VCAM-1. Up to the concentration of 50 μg/mL SiNPs, the protein levels of ICAM-1 and VCAM-1 were decreased significantly compared to that of control group, respectively. In addition, the results showed that SiNPs had a strong inhibitory effect on the expression of VACM-1 while a modest inhibitory effect on ICAM-1 expression. However, the E-selectin was not detectable in any treatment groups, indicating that SiNPs had no effect on E-selectin expression.

### Crosstalk between autophagy and angiogenesis signaling pathways

**Figure 7:**
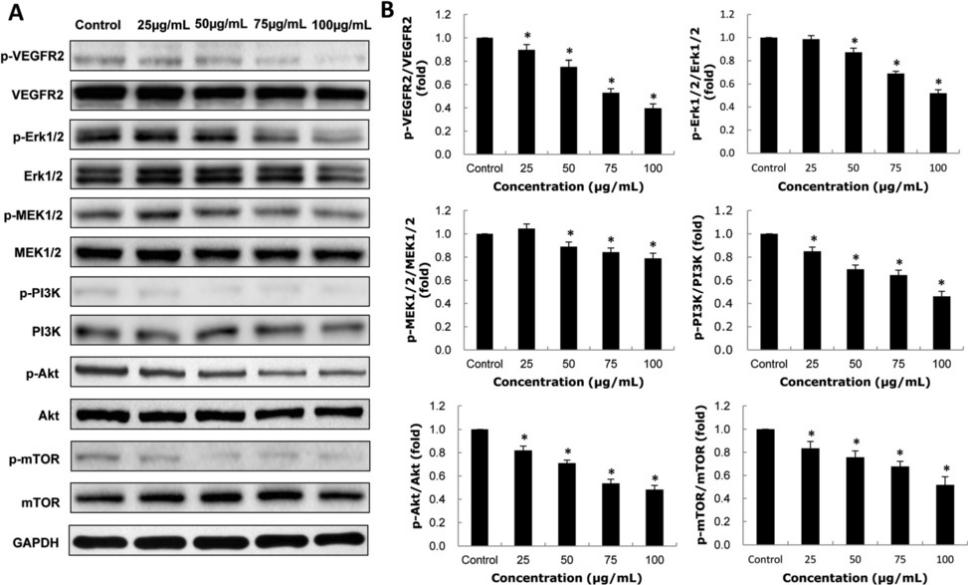
**Effects of SiNPs on autophagy and angiogenesis signaling pathways. (A)** Effects of SiNPs on the expression of p-VEGFR2, VEGFR2, p-MEK1/2, MEK1/2, p-Erk1/2, Erk1/2, p-PI3K, PI3K, p-Akt, Akt, p-mTOR, mTOR proteins. GAPDH was used as an internal control to monitor for equal loading. **(B)** SiNPs induced autophagy through the up-regulation of MAPK/Erk1/2/mTOR signaling and PI3K/Akt/mTOR signaling pathways; while SiNPs inhibited angiogenesis via the down-regulation of VEGFR2/MEK1/2/Erk1/2 and VEGFR2/PI3K/Akt signaling pathways. Data are expressed as means ± S.D. from five independent experiments (*p < 0.05).

**Figure 8:**
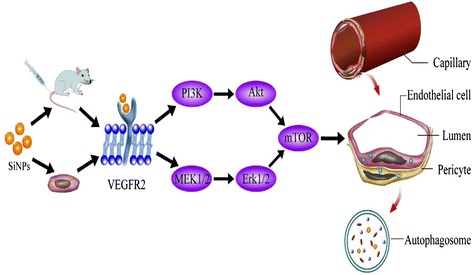
**Schematic model of the molecular mechanisms on VEGFR2-mediated crosstalk between autophogy and angiogenesis signaling pathways triggered by SiNPs.**

As shown in Figure 7, the ratios of p-VEGFR2/VEGFR2, p-MEK1/2/MEK1/2, p-Erk1/2/Erk/1/2, p-PI3K/PI3K, p-Akt/Akt, and p-mTOR/mTOR in HUVECs were significantly decreased in a dose-dependent manner after exposure to SiNPs for 24 h. Our data showed that SiNPs activated autophagy via the suppression of MEK1/2/Erk1/2/mTOR and PI3K/Akt/mTOR signaling pathways; while SiNPs inhibited angiogenesis through the down-regulation of VEGFR2/MEK1/2/Erk1/2 signaling and VEGFR2/PI3K/Akt signaling pathways. Interestingly, there was a crosstalk between the autophagy pathway and the angiogenesis pathway via VEGFR2. In addition, the relative densitometric analysis of the proteins showed that there was no significant difference at the ratios of p-MEK1/2/MEK1/2 and p-Erk1/2/Erk/1/2 in the low dosage group (25 μg/mL); whereas the ratios of p-VEGFR2/VEGFR2, p-PI3K/PI3K, p-Akt/Akt, and p-mTOR/mTOR were significantly difference in the 25 μg/mL group compared to that of control group. Thus, our findings suggested that the VEGFR2/PI3K/Akt/mTOR signaling had a dominant role in the SiNPs-induced activation of autophagy and the inhibition of angiogenesis. A schematic model of the molecular mechanisms on VEGFR2-mediated crosstalk between autophogy and angiogenesis signaling pathways triggered by SiNPs was presented in Figure 8.

## Discussion

Although effect of ENMs on the cardiovascular system is gaining attention, there is still a lack of relevant studies in vitro and in vivo. In this report, we focused on the biological behavior of SiNPs on cardiovascular systems, and illustrated for the first time the influence of SiNPs-induced autophagy on endothelial cell homeostasis and angiogenesis in vitro and in vivo. It might provide a better understanding of ENMs-based environmental safety and the toxicity mechanism of cardiovascular system, especially on IHD.

Currently, autophagy is being considered as one of emerging mechanism of nanomaterial. However, detailed mechanisms of nanomaterials-induced autophagy have not been fully investigated. Most recent studies focus on nanotoxicity using in vitro models with a wide range of cell lines [26-28]; only a few studies have been reported nanoparticles as a novel class of autophagy activators in vivo [29,30]. Ultrastructural analysis has been recommended as the golden standard of autophagy diagnosis [31], and the TEM observation showed that autophagy was activated by SiNPs in the vascular endothelium (Figure 2). It was reported that the ribonucleic acid (RNA) component of ribosomes was both necessary and sufficient for autophagy activation through the conjugation reaction of the autophagy related genes ATG12-ATG5 [32], the numerous free ribosomes observed in our study may have contributed to SiNPs-induced autophagy. Surprisingly, several autophagic vacuoles and a number of membrane-bound SiNPs were present in pericytes. Our findings indicated that the SiNPs could be internalized into the pericytes as well as inducing autophagic activity. It is well known that the endothelium is surrounded by pericytes that communicate with each other. The pericytes-endothelial cells interactions directly modulate microvascular development, stabilization, maturation, and remodeling [33]. Moreover, the interaction of pericytes-endothelial cells contributes to angiogenesis [34]. The process of angiogenesis depends on the multiplicity of cytokines, complexity of pathways and high coordination between endothelial cells and pericytes [35]. Thus, we hypothesized that the interaction of SiNPs-induced autophagy in pericytes-endothelial cells might play an important role in angiogenesis. Although this interaction is recognized as a critical factor in microvascular homeostasis, up to now, researchers pay less attention to pericytes than endothelial cells. Thus, more studies are needed to clarify the interaction mechanisms of pericytes-endothelial cells.

It has been demonstrated that angiogenic growth factors and their respective receptors were expressed in normal tissue [36]. The vascular endothelial growth factor (VEGF) bound with the receptor tyrosine kinases VEGFR2, mediating its biological effects on angiogenesis [37]. Our result showed that the VEGFR2 positive cells were decreased gradually in all SiNPs-treated groups (Figure 3). It was reported that efficient inhibition of the VEGF/VEFGR2 pathway could lead to ischaemic cardiomyopathy and ultimately died of cardiac failure in mice model [36]. In this study, LC3, an autophagosome marker protein, was mainly expressed on the vascular endothelium rather than cardiomyocytes in a dose-dependent manner (Figure 3). Meanwhile, there was no marked change of histomorphology in the heart tissue, as well as no detected TUNEL positive cells in all treatment groups (Additional file 1: Figure S1), which indicated that the cell death (apoptosis and necrosis) of vascular endothelium was not responsible for the impaired angiogenesis under this experimental condition. Taken together, these results demonstrated that SiNPs can induce autophagy and attenuate angiogenesis, but had little effect on the apoptosis and necrosis in vascular endothelium.

Cell adhesion has played a critical role in regulating a series of cellular functions, such as cell growth, migration, and differentiation [38]. Up-regulation of cellular adhesion molecules is considered to be a prerequisite for angiogenesis. During the pathological progression of diseases, the activation of endothelial adhesion molecules is involved in promoting angiogenesis, atherosclerosis and tumor growth [39]. In the present study, our results showed that SiNPs could decrease the expression of ICAM-1 and VCAM-1; whereas SiNPs had no obvious effect on E-selectin expression (Figure 4 and Additional file 1: Figure S1). Based on these results, we could infer that a coping mechanism exists when dealing with nanomaterials in vivo; the down-regulation of cellular adhesion molecules and the following inhibition of angiogenesis occurred to maintain vascular homeostasis. The findings obtained in our study may provide a clue and correlation for IHD and cardiovascular toxicities triggered by nano-sized particles.

We previously reported that the SiNPs were internalized into cells through the endocytiosis, which is the most common pathway involved in nanomaterial cellular uptake [17,40]. When the SiNPs entered into cells, it initially contacted with the cytoskeleton organization. The cytoskeleton, composed of microtubules, intermediate filaments and microfilaments (actin) as the internal scaffold of cells, exhibits a special assembly to protect cell stability [38]. In eukaryotic cells, the microfilaments that contain F-actin proteins are the most elastic and diversified element in the cytoskeleton, which contributes to cell morphology, endocytosis, dynamics and adhesion [41]. The SiNPs were internalized into HUVECs and disturbed the cytoskeleton in a dose-dependent manner (Figure 5). It was reported that actin is one of the most commonly bound proteins with nano- or micro-particles [42]. Thus, the states of actin polymerization and depolymerisation caused by SiNPs might be one of the toxic mechanisms.

There is a growing body of literatures linking cytoskeleton organization with autophagy: in the process of actin remodelling, the histone deacetylase-6 was related to the fusion of autophagosome and lysosome [43]; a recent report found that the cytoskeleton was mediated in autophagic activity via the regulation of septins [44]. Our results from the ultrastructural analysis showed that SiNPs induced mitochondrial damage and autophagic vacuoles accumulation in endothelial cells (Figure 6). The internalized SiNPs could attack mitochondria directly, or cause mitochondrial damage indirectly via oxidative stress [45]. The primary feature of mitochondria is efficient coupling of cellular respiration to ATP production. However, since the vascular system does not require much energy, the mitochondria function is more of a responsive sensing system (RSS) than a simply energy production system [46]. SiNPs-induced mitochondrial depolarization was further evaluated by the loss of MMP in HUVECs (Additional file 1: Figure S2). MMP is a quality control for mitochondria, whereas the hyperpolarized mitochondria can improve the organelles network; and the depolarized mitochondria are eliminated by autophagy or mitophagy (mitochondrial specific autophagy) [47]. We also confirmed the SiNPs induced autophagy by the standard marker protein LC3, which usually exhibiting a molecular form conversion from cytosolic LC3-I into its enzymatic counterpart LC3-II during autophagy [48] (Additional file 1: Figure S3). And we previously used the autophagy inhibitor 3-Methyladenine (3-MA) to verify the inhibition of autophagy induced by SiNPs [24]. Because vasculature plays a key role in delivering oxygen rather than consuming oxygen, mitophagy is of great importance in vascular biology. To minimize oxygen consumption, one potential mechanism is to reduce the amount of mitochondria by increasing their elimination via mitophagy. Therefore, it is not surprising that the composition of mitochondria in endothelial cells is 5%; while in hepatic cells, the composition of mitochondria is as much as 28% [46]. Yet, autophagy acts as a double-edged sword. Excess induction of autophagy, either from the upregulated or blocked autophagy flux, will result in toxic effects on the vascular system. If the reduction of mitochondria is below the base threshold value of endothelial cells, the adaptability for maintaining intracellular homeostasis will gradually disappear and will actually lead to a change in the function of the endothelial cells [49].

Additionally, it is well documented that the disruption of the actin cytoskeleton can directly alter the capacity of cell adhesion [41]. The primary contractile construction in numerous non-muscle cells are stress fibers that contain F-actin and myosin II and regulate the mechanotransduction with focal adhesion [41]. Phosphorylation of myosin light chains mediates the formation of actin stress fibers, which are associated with cell contraction and cell adhesion [50]. In this study, SiNPs decreased the expression of ICAM-1 and VCAM-1 in a dose-dependent manner; whereas no detectable level of E-selectin protein was observed in any of the SiNPs-treated groups (Additional file 1: Figure S4). Similar with our findings, using the cytoskeleton inhibitors Y27632 and ML-7, VandenBerg et al. confirmed that the expression of VCAM-1 and ICAM-1 in endothelial cells was dependent on cycling of the actin cytoskeleton, except E-selectin [51]. This could account for the inhibitory effect that SiNPs had on ICAM-1 and VCAM-1, but not on E-selectin in vivo and in vitro. The detailed mechanism needed to be further explored.

Finally, we analyzed the protein expression of autophagy and angiogenesis signaling pathways in SiNPs-treated HUVECs (Figure 7). Autophagy, as a major protein degradative process for maintaining cellular homeostasis, affects various human processes including cancer, neurodegeneration and cardiovascular disorders [52]. An increasing body of autophagy regulators has been confirmed in recent years. The classical signaling pathways for regulating mammalian autophagy relates to the serine/threonine kinase mTOR, which is mainly mediated by PI3K/Akt and MAPK/Erk1/2 signaling transduction [53]. PI3K, Akt, MEK1/2 and Erk1/2 are also generally thought to be involved in VEGFR2-dependent angiogenesis. Up-regulation of VEGFR2 activates the downstream signaling pathways, which facilitates cell growth, survival, differentiation and pro-angiogenesis in preexisting vasculature [54]. In this study, western blot analysis clearly showed a crosstalk between the autophagy and the angiogenesis pathway via the down-regulation of VEGFR2 triggered by SiNPs. Moreover, our results showed that the VEGFR2/PI3K/Akt/mTOR signaling played an important role in the involved mechanisms. Based on the results above, a schematic model of the molecular mechanisms on the crosstalk between SiNPs-induced autophogy and the angiogenesis pathway is present in Figure 8. Further investigations are needed to determine whether the crosstalk between SiNPs-induced autophogy and angiogenesis found in our study is a common mechanism for most nanoparticles.

## Conclusions

Our finding indicates that the cardiovascular toxicity triggered by SiNPs occurs mainly in vascular endothelium rather than cardiomyocytes. The SiNPs can disrupt the cytoskeleton organization, activate autophagic activity in endothelial cells and percytes, cause mitochondria damage, and attenuate the expression of cellular adhesion molecules, which contribute to the disturbance of the endothelial cell homeostasis, and eventually impair angiogenesis. VEGFR2/PI3K/Akt/mTOR and VEGFR2/MAPK/Erk1/2/mTOR signaling pathway are involved in SiNPs-induced cardiovascular toxicity. Moreover, there is a crosstalk between the VEGFR2-mediated autophagy signaling and angiogenesis signaling pathways. Our findings provide laboratory evidence for the potential mechanisms of ultrafine fractions on cardiovascular diseases, which may help to evaluate the hazardous effect of nano-sized particles on cardiovascular system.

## Materials and methods

### SiNPs preparation and characterization

SiNPs were prepared using the Stöber method and fully characterized in our previous studies [23,25,55]. Briefly, 2.5 mL of tetraethylorthosilicate (TEOS) (Sigma, USA) was added to premixed ethanol solution (50 mL) containing ammonia (2 mL) and water (1 mL). The reaction mixture was kept at 40°C for 12 h with continuous stirring (150 r/min). The resulting particles were isolated by centrifugation (12,000 r/min, 15 min) and washed three times with deionized water and then dispersed in 50 mL of deionized water. The size of SiNPs was measured by TEM (JEOL JEM2100, Japan), and the size distribution was analyzed using ImageJ software (National Institutes of Health, USA). The hydrodynamic sizes and zeta potential of SiNPs were examined by Zetasizer (Malvern Nano-ZS90, Britain). Suspensions of SiNPs were dispersed by sonicator before use (160 W, 20 kHz, 5 min) (Bioruptor UDC-200, Belgium).

### Animal experiment

Male and female ICR mice (8 weeks old and 20–22 g in body weight) were purchased from Weitong-Lihua Experimental Animal Center (Beijing, China). They were separated by sex in plastic cages with stainless steel mesh lids in a ventilated room. The room was maintained at 20 ± 2°C and 60 ± 10% relative humidity with a 12 h light–dark cycle. The mice were fed with water and sterilized food. Prior to treatment, the mice were not fed overnight. All animal care and experiments were approved by the Animal Ethics Committee at Capital Medical University (approval number 2011-X-072). A series dosage of SiNPs (0, 29.5, 103.5 and 177.5 mg/kg) was set based on the LD_50_ estimating value (262.45 ± 33.78 mg/kg) from SiNPs acute exposure by intravenous injection [23]. Sterile physiological saline was injected to the mice as a control. The acute toxicity was evaluated at 14 d after intravenous injection. At the end of the experiment, all animals were sacrificed for subsequent research.

### Cell culture experiment

HUVECs were purchased from the Cell Resource Center, Shanghai Institutes for Biological Sciences (SIBS, China). The cells were maintained in DMEM (Gibco, USA) supplemented with 10% fetal bovine serum (Gibco, USA), 100 U/mL penicillin and 100 μg/mL streptomycin, and cultured at 37°C in 5% CO_2_ humidified environment. For experiments, the cells were seeded in 6-well plates at a density of 1 × 10^5^ cells/mL and allowed to attach for 24 h, then treated with SiNPs (25, 50, 75 and 100 μg/mL) suspended in DMEM for 24 h. Controls were supplied with an equivalent volume of DMEM without SiNPs. Each group had five replicate wells.

### Histopathology

The heart samples were removed and fixed in 10% formalin, embedded in paraffin, sectioned, and stained with hematoxylin and eosin (HE) for histological examination according to the standard techniques. After staining, the slides were observed and examined by optical microscope (Olympus X71-F22PH, Japan).

### Apoptosis assay

Heart sections were stained and analyzed by TUNEL assay to detect the locus of apoptotic cells according to the manufacturer’s protocol (KeyGen, China). Cells with brown nuclear staining can be viewed as positive. TUNEL-positive cells were carefully evaluated under double-blind conditions. Photos were taken by optical microscope (Olympus X71-F22PH, Japan).

### Immunohistochemistry

After deparaffinisation and rehydration, the paraffin embedded heart sections were placed in a 10 mM citrate buffer solution and treated with 3% H_2_O_2_ in PBS for 5 min. Then, the sections were blocked with 10% normal goat serum for 10 min, and incubated overnight at 4°C with primary antibody [VEGFR2, VCAM-1, ICAM-1, E-selectin (Abcam, Britain), LC3 (CST, USA)] or an equivalent amount of normal goat IgG (CST, USA) as a negative control. After treated with avidin-biotin affinity system for 30 min at room temperature, and stained with 3-3’ diaminobenzidine substrate, the sections were examined under a optical microscope (Olympus X71-F22PH, Japan).. All positive cells were carefully evaluated under double-blind conditions. Image-pro Plus software (Media Cybernetics, United States) was used to calculate the average integrated optical density (IOD) per stained area (μm^2^) (IOD/area) for positive staining.

### TEM observation of Autophagy

Briefly, the samples was collected and immediately fixed overnight in 3% glutaraldehyde. Then the samples were rinsed three times with 0.1 M PB and postfixed with 1% osmic acid for 2 hours. After being rinsed three times with 0.1 M PB and serially dehydrated with 50%, 70%, 80%, 90% and 100% alcohol and 100% acetone, the samples were embedded in epoxy resin for making the blocks of cells or tissues. The ultrathin sections (50 nm) were obtained by an ultramicrotome (Ultracut UCT, Leica, Germany). They were then stained with lead citrate and uranyl acetate, and detected by TEM (JEM2100, JEOL, Japan).

### Cell cytoskeleton staining

HUVECs were seeded at 1 × 10^4^ cells in 35 mm-diameter glass bottom cell culture dish and were cultured in DMEM as mentioned above. After 24 h of cell attachment, the cells were treated with Ruthenium (II) hydrate (Ru(phen)_3_^2+^) interior-labeled silica nanoparticles (50 μg/mL) for 24 h at 37°C in serum-free medium. These red fluorescent silica nanoparticles were prepared and characterized by a modified Stöber method as described previously [56]. Cells were then washed several times with PBS and fixed with 4% paraformaldehyde at room temperature for 10 min. Cells were then washed 3 times with PBS and fixed with 4% paraformaldehyde at room temperature for 10 min. The cells were washed with 0.1% Triton X-100 three times and incubated with Phalloidin-FITC Actin-Tracker Green (Jiancheng, China) at room temperature for 30 min. The Actin-Tracker was dissolved in the mixture of 0.1% Triton X-100 and 3% bovine serum albumin (BSA) (Sigma, USA) for staining the F-actin. After that, the nucleus was stained with 5 μg/mL 4,6-diamidino-2-phenylindole (DAPI) (Sigma, USA) in PBS for 5 min. Cellular uptake and F-actin formation were observed by LSCM (Leica TCS SP5, Germany).

### Detection of MMP

MMP was detected by using the fluorescent probe JC-1 (Sigma, USA). This probe can selectively enter into mitochondria and reversibly change color from red to green as the membrane potential decreased. The ratio of green to red expresses the change of MMP. Cells were treated with SiNPs (25, 50, 75, and 100 μg/mL) for 24 h. After washing with PBS, the cells were incubated with 10 μg/mL working solution of JC-1 for 20 min. Then the cells were washed with PBS twice and analyzed by flow cytometry (FCM) (Becton-Dickison, USA). The green fluorescence intensity was determined at an excitation wavelength of 488 nm and an emission wavelength of 525 nm, whereas the red fluorescence intensity was determined at an excitation wavelength of 488 nm and an emission wavelength of 590 nm. For each sample, at least 1 × 10^4^ cells were collected.

### Western blot analysis

Total cellular protein extracts were determined by using the bicinchoninic acid (BCA) protein assay (Pierce, USA). The equal amount of lysate proteins (40 μg) was loaded onto SDS-polyacrylamide gels (12% separation gels) and electrophoretically transferred to polyvinylidene fluoride (PVDF) membranes (Millipore, USA). After blocking with 5% nonfat milk in Tris-buffered saline (TBS) containing 0.05% Tween-20 (TBST) for 1 h at room temperature, the membrane was incubated with phosphoinositide 3-kinase (PI3K), p-PI3K, protein kinase B (PKB or Akt), p-Akt, mammalian target of rapamycin (mTOR), p-mTOR, mitogen-activated protein kinase (MEK1/2), p-MEK1/2, extracellular regulated protein kinase (Erk1/2), p-Erk1/2, LC3 (CST, USA), VEGFR2, p-VEGFR2, ICAM-1, VCAM-1, E-selectin antibodies (Abcam, Britain) (1:1000, rabbit antibodies) overnight at 4°C, washed with TBST, and incubated with a horseradish peroxidase-conjugated anti-rabbit Ig G secondary antibody (CST, USA) for 1 h at room temperature. After being washed three times with TBST, the antibody-bound proteins were detected using the Enhanced Chemiluminescence (ECL) (Pierce, USA). Densitometric analysis of the western blot was performed using Image Lab™ Software (Bio-Rad, USA).

### Statistical analysis

Data were expressed as mean ± S.D. and significance was determined by using one-way analysis of variance (ANOVA) followed by least significant difference (LSD) test to compare the differences between groups. Differences were considered significant at p < 0.05.

## Additional file

Additional file 1: Table S1 Hydrodynamic size and Zeta potential of SiNPs in dispersion media. **Figure S1.** Effect of SiNPs on histomorphology, apoptosis and E-selectin in ICR mice heart tissue sections. **Figure S2.** Effect of SiNPs on mitochondrial membrane potential (MMP) in HUVECs. **Figure S3.** LC3-I/LC3-II conversion in SiNPs-treated HUVECs. **Figure S4.** Effects of SiNPs on cellular adhesion molecule expression in HUVECs.
