# Supplementary material for: Silica nanoparticles enhance autophagic activity, disturb endothelial cell homeostasis and impair angiogenesis
Source: Part Fibre Toxicol. 2014 Sep 30;11:50. doi: 10.1186/s12989-014-0050-8 (PMC4193984; doi:10.1186/s12989-014-0050-8)
Supplement: Additional file 1: Table S1 — Hydrodynamic size and Zeta potential of SiNPs in dispersion media. Figure S1. Effect of SiNPs on histomorphology, apoptosis and E-selectin in ICR mice heart tissue sections. Figure S2. Effect of SiNPs on mitochondrial membrane potential (MMP) in HUVECs. Figure S3. LC3-I/LC3-II conversion in SiNPs-treated HUVECs. Figure S4. Effects of SiNPs on cellular adhesion molecule expression in HUVECs. [file 12989_2014_50_MOESM1_ESM.docx]

**Additional file 1: Table S1**. Hydrodynamic size and Zeta potential of SiNPs in dispersion media

|  | Distilled water | |  | Physiological saline | |  | DMEM | |
| --- | --- | --- | --- | --- | --- | --- | --- | --- |
|  | Diameter  (nm) | Zeta potential (mV) |  | Diameter  (nm) | Zeta potential  (mV) |  | Diameter  (nm) | Zeta potential (mV) |
| 1h | 109.02 ± 3.54 | -43.51 ± 4.67 |  | 110.26 ± 4.81 | -39.44 ± 3.69 |  | 108.01 ± 1.43 | -40.13 ± 4.29 |
| 3h | 107.92 ± 3.31 | -40.20 ± 2.13 |  | 108.93 ± 2.79 | -37.26 ± 2.50 |  | 109.22 ± 3.10 | -38.62 ± 2.76 |
| 6h | 108.10 ± 2.23 | -42.53 ± 3.84 |  | 107.64 ± 2.33 | -40.30 ± 2.47 |  | 107.51 ± 2.07 | -40.05 ± 3.60 |
| 12h | 106.34 ± 1.67 | -43.11 ± 4.06 |  | 105.42 ± 3.01 | -38.11 ± 3.45 |  | 106.27 ± 4.09 | -38.74 ± 2.91 |
| 24h | 105.79 ± 2.51 | -44.45 ± 3.71 |  | 105.65 ± 2.18 | -37.70 ± 3.02 |  | 105.70 ± 2.44 | -39.10 ± 2.83 |

Data are expressed as means ± S.D. from five independent experiments.


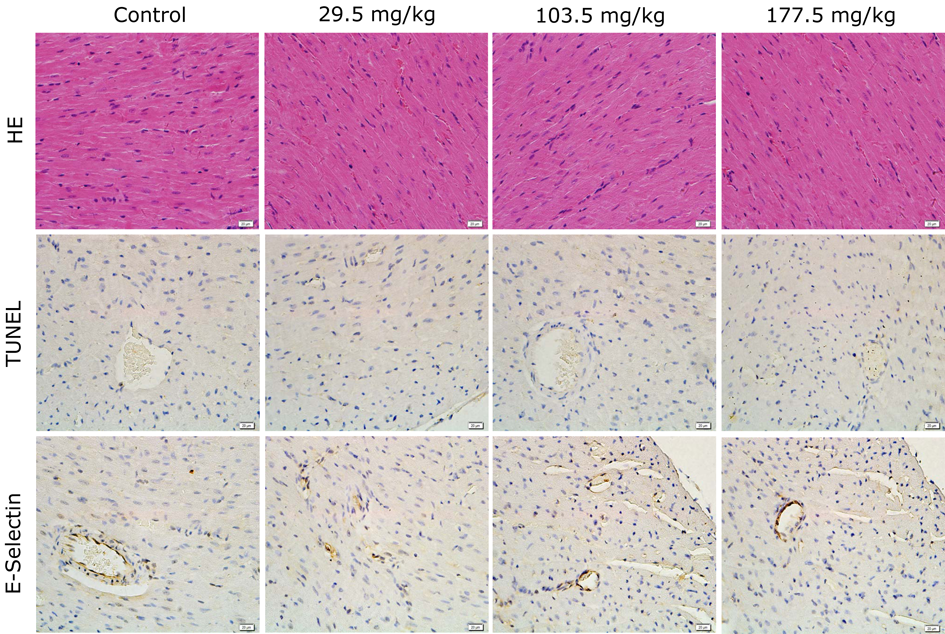


**Additional file 1: Figure S1**. Effect of SiNPs on histomorphology, apoptosis and E-selectin in ICR mice heart tissue sections. The histopathological examination was performed with hematoxylin and eosin (HE); Detection of apoptotic cells was measured by TUNEL assay; Expression of E-selectin was stained by immunohistochemistry.


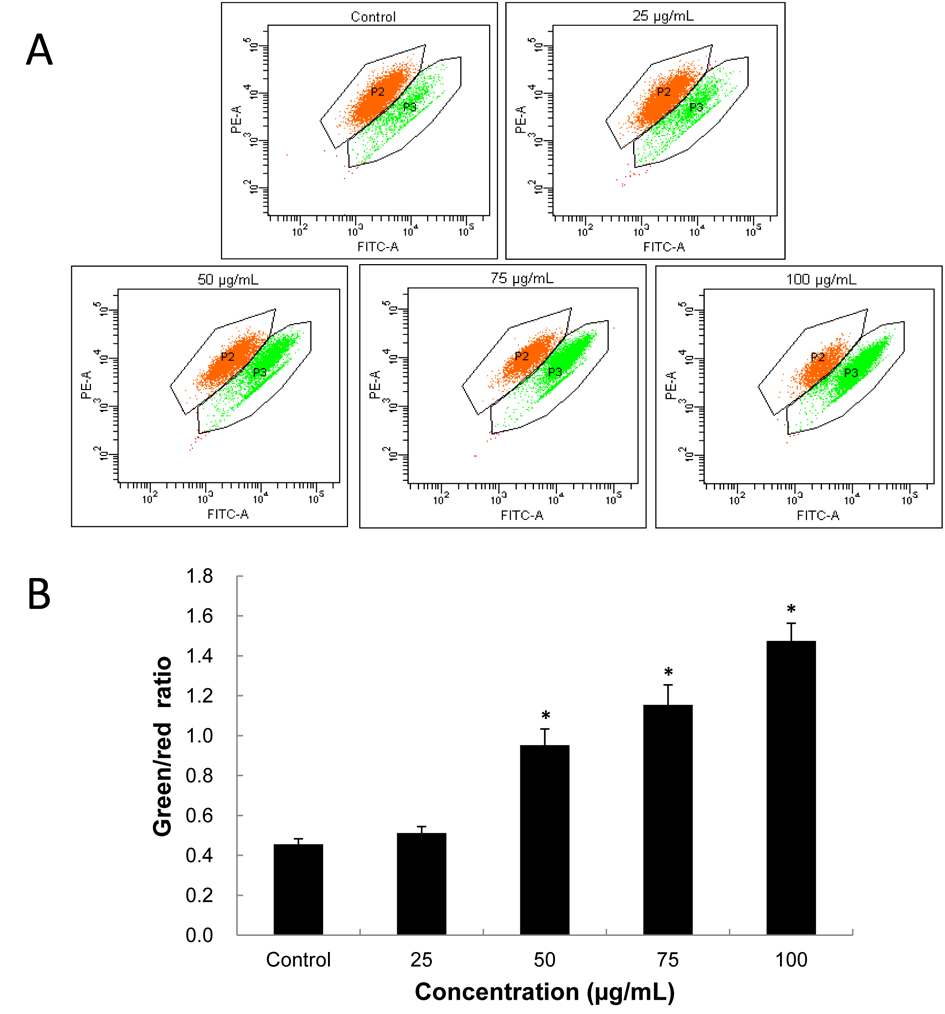


**Additional file 1: Figure S2**. Effect of SiNPs on mitochondrial membrane potential (MMP) in HUVECs. The MMP was detected with JC-1 probe by flow cytometry (A). The green/red fluorescence intensity ratio was used to express the changes of MMP and the increased ratio indicates decrease of MMP (B). Data are expressed as means ± S.D. from five independent experiments (*p＜0.05).


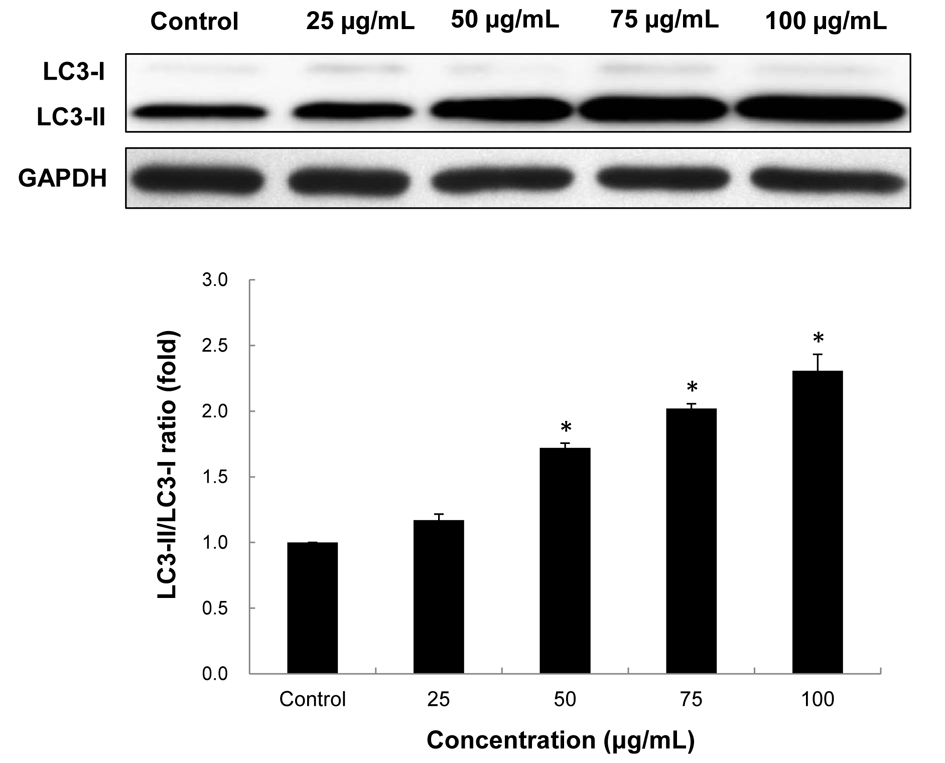


**Additional file 1: Figure S3**. LC3-I/LC3-II conversion in SiNPs-treated HUVECs. Relative densitometric analysis showed that the ratio of LC3-II/LC3-I significantly elevated in a dose-dependent manner. Data are expressed as means ± S.D. from five independent experiments (*p＜0.05).


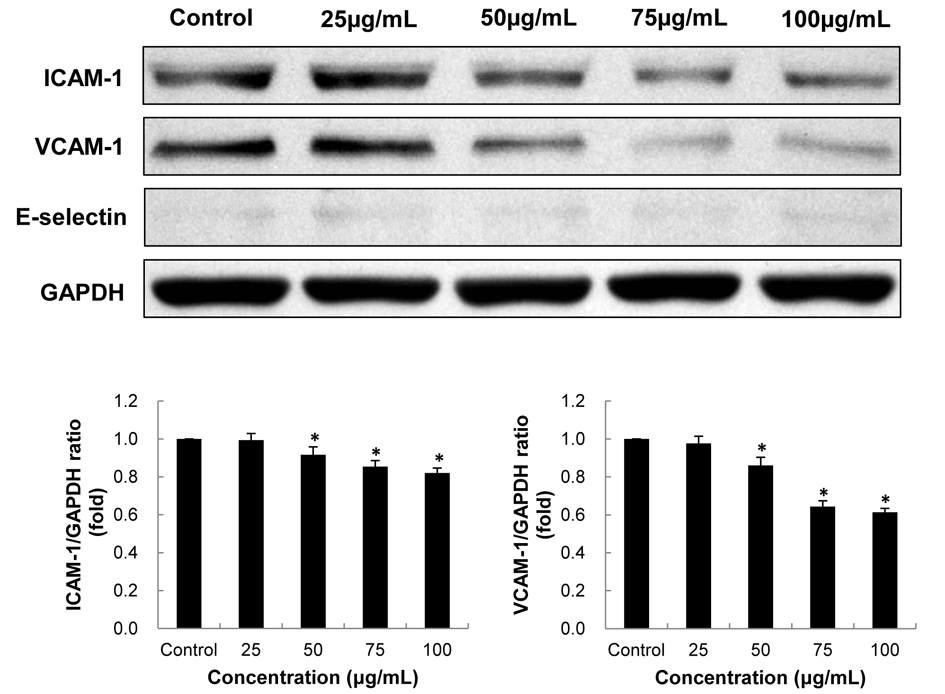


**Additional file 1: Figure S4**. Effects of SiNPs on cellular adhesion molecule expression in HUVECs. The SiNPs had an inhibitory effect on the expression of VACM-1 and ICAM-1, but not on E-selectin expression. Data are expressed as means ± S.D. from five independent experiments (*p＜0.05).
